# Unraveling the interplay between norovirus infection, gut microbiota, and novel antiviral approaches: a comprehensive review

**DOI:** 10.3389/fmicb.2023.1212582

**Published:** 2023-07-06

**Authors:** Geng-Hao Bai, Meng-Chen Tsai, Sheng-Chieh Lin, Yi-Hsiang Hsu, Shih-Yen Chen

**Affiliations:** ^1^Department of Internal Medicine, National Taiwan University Hospital, College of Medicine, National Taiwan University, Taipei, Taiwan; ^2^Department of General Medicine, Taipei Medical University Hospital, Taipei, Taiwan; ^3^Department of Pediatrics, School of Medicine, College of Medicine, Taipei Medical University, Taipei, Taiwan; ^4^Department of Pediatrics, Division of Allergy, Asthma and Immunology, Shuang Ho Hospital, New Taipei, Taiwan; ^5^Beth Israel Deaconess Medical Center and Harvard Medical School, Boston, MA, United States; ^6^Broad Institute of MIT and Harvard, Cambridge, MA, United States; ^7^Department of Pediatrics, Division of Pediatric Gastroenterology and Hepatology, Shuang Ho Hospital, New Taipei, Taiwan; ^8^TMU Research Center for Digestive Medicine, Taipei Medical University, Taipei, Taiwan

**Keywords:** norovirus, microbiota, probiotics, norovirus vaccine, human intestinal enteroid

## Abstract

Norovirus infection is a leading cause of acute gastroenteritis worldwide and can also cause harmful chronic infections in individuals with weakened immune systems. The role of the gut microbiota in the interactions between the host and noroviruses has been extensively studied. While most past studies were conducted *in vitro* or focused on murine noroviruses, recent research has expanded to human noroviruses using *in vivo* or *ex vivo* human intestinal enteroids culture studies. The gut microbiota has been observed to have both promoting and inhibiting effects on human noroviruses. Understanding the interaction between noroviruses and the gut microbiota or probiotics is crucial for studying the pathogenesis of norovirus infection and its potential implications, including probiotics and vaccines for infection control. Recently, several clinical trials of probiotics and norovirus vaccines have also been published. Therefore, in this review, we discuss the current understanding and recent updates on the interactions between noroviruses and gut microbiota, including the impact of norovirus on the microbiota profile, pro-viral and antiviral effects of microbiota on norovirus infection, the use of probiotics for treating norovirus infections, and human norovirus vaccine development.

## 1. Introduction

Acute gastroenteritis is the second most burdensome infectious disease worldwide. Norovirus, a key pathogen associated with nearly one-fifth of all cases of acute gastroenteritis (Ahmed et al., 2014), is responsible for 18% of diarrheal disease globally, affecting both high-and low-income countries alike. Norovirus causes approximately 200,000 deaths annually worldwide, with over 70,000 of these deaths occurring among children in developing countries (Lopman, 2015; Centers for Disease Control and Prevention, 2023). Additionally, norovirus is responsible for 58% of foodborne illnesses in the United States, costing approximately $2 billion in lost productivity each year. Noroviruses belong to the *Caliciviridae* family and are non-enveloped, single-stranded RNA viruses. In 2019, the classification of noroviruses was updated, with 10 genogroups (GI-GX) and 48 genotypes [9 GI, 27 GII, 3 GIII, 2 GIV, 2 GV, 2 GVI, and 1 genotype each for GVII, GVIII, GIX (formerly GII.15), and GX] (Chhabra et al., 2019). Norovirus GII genotype 4 (GII.4) variants have been the predominant strains worldwide for decades (White, 2014), but recent studies have reported the emergence of GII.17 variants causing significantly increased outbreaks of acute gastroenteritis (Jin et al., 2016; Lu et al., 2016; Cheung et al., 2019). The presence of histo-blood group antigens (HBGAs) on the intestinal epithelium is essential for human norovirus infection, and GII.17 norovirus causing outbreak infections has been shown to have a wide spectrum of HBGAs susceptibility (Chan et al., 2015; Zhang et al., 2015). In addition, the gut microbiota has been found to play a role in norovirus infection with pro-viral and antiviral effects on both human and murine noroviruses (Sullender and Baldridge, 2018; Walker and Baldridge, 2019; Pena-Gil et al., 2021; Soorneedi and Moore, 2022). Recent years have seen an increase in *in vitro* trials using cell lines, *in vivo* clinical trials in animals and humans, and successful cultivation of human norovirus in *ex vivo* human intestinal enteroid (HIE) cultures (Ettayebi et al., 2016). Understanding the potential promotive or inhibitory role of the microbiota, the application of probiotics, and vaccine development in norovirus infection remains an area of active interest. Further understanding of the role of the microbiota in norovirus infection could provide insights into human norovirus pathogenesis and aid in the development of antiviral strategies. Therefore, the purpose of this review is to investigate and update the impact of norovirus on the gut microbiota profile, the role of the microbiota in norovirus infection, the use of probiotics for norovirus infections, human norovirus vaccine development.

## 2. Histo-blood group antigens and human milk oligosaccharides

Glycan interaction plays a crucial role in norovirus infection. Glycan epitopes have been found on histo-blood group antigens (HBGAs) and human milk oligosaccharides (HMOs) (Etzold and Bode, 2014). HBGAs are soluble antigens present in saliva and expressed on the mucosal epithelium of the digestive tract. HBGAs are catalyzed by a set of glycosyltransferases encoded by three major gene families: secretor, Lewis, and ABO. Oligosaccharides containing Lewis and ABH antigenic epitopes are involved in the variable binding activity with norovirus (Huang et al., 2003). The Secretor (*Se*) gene codes for an α-1,2 fucosyltransferase (FUT2), the Lewis (*Le*) gene codes for an α-1,3 or α-1,4 fucosyltransferase (FUT3), while the ABO family codes for two glycosyltransferases (A and B enzymes) (Tan and Jiang, 2014). Moreover, the FUT2 gene, which synthesizes the H-type 1 antigen in saliva and mucosa, is associated with susceptibility to norovirus infections. Individuals who express a functional FUT2 enzyme (known as secretor status) are more susceptible to norovirus infections (Nordgren and Svensson, 2019), while those who do not express a functional FUT2 enzyme (known as non-secretors) like the FUT2 non-secretor (*se428se428*) genotype are resistant to nosocomial and sporadic outbreaks of norovirus (Thorven et al., 2005). Clinical studies have shown that individuals with a homozygous recessive inactivating G428A mutation of the FUT2 gene, who do not express the H type-1 oligosaccharide ligand for norovirus binding, may be genetically resistant to norovirus infection (Lindesmith et al., 2003). Another clinical study showed that GII.4 strains of human norovirus exclusively infect secretors (Currier et al., 2015). Moreover, human noroviruses have a preference to certain types of HBGAs. Observational studies have also shown that individuals with blood group O (H antigen) are more likely to be infected with norovirus, while individuals with blood group B appear to have reduced susceptibility to symptomatic norovirus infection (Hutson et al., 2002; Hennessy et al., 2003).

Human milk oligosaccharides (HMOs) are complex carbohydrates synthesized in the breast gland and are abundant in human milk. The amount and composition of HMOs vary highly between women, and each structurally defined HMO may have distinct functionality. HMOs directly or indirectly modulate the infant’s physiological systems (Zhang S. et al., 2021). Firstly, since HMOs are not digested by the infant and serve as metabolic substrates for select microbes, they can shape the infant gut microbiome. Next, HMOs are soluble decoy receptors that block the attachment of viral, bacterial, or protozoan parasite pathogens to epithelial cell surface sugars, which may prevent infectious diseases in the gastrointestinal tract. HMOs are also antimicrobials that act as bacteriostatic or bacteriocidal agents. In addition, HMOs alter host epithelial and immune cell responses with potential benefits for the neonate (Bode, 2015). The presence and quantity of HMOs are also related to the secretor status and the Lewis group type. FUT2-dependent HMOs include 2′-fucosyllactose (2’FL), difucosyllactose (DFLac), and lactose-N-fucopentaose (LNFP) I. Non-secretor mothers have been found to produce significantly less HMOs compared to secretor mothers due to the absence of 2’FL, which accounts for a high proportion in secretor milk (Azad et al., 2018). Unlike HBGAs, HMOs provide protection against norovirus infection by competing with the HBGAs binding site on the norovirus capsid (Jiang et al., 2004; Shang et al., 2013). HMOs such as 2’FL and 3′-fucosyllactose (3’FL) could block norovirus from binding to HBGAs (Weichert et al., 2016), and 2′FL and 3’FL might function as broadly reactive antivirals against multiple norovirus genogroups (Koromyslova et al., 2017; Lalithamaheswari and Anu Radha, 2022).

## 3. Human intestinal enteroids

To address the challenges in evaluating the role of gut microbiota on norovirus infection, a recent advancement is the establishment of the human intestinal enteroids (HIEs) system. HIEs provide a valuable tool for studying norovirus replication (Cates et al., 2020). Estes et al. (2019) reviewed the applications of HIEs, which include studying virus-specific replication requirements, evaluating human host-pathogen interactions, and supporting pre-clinical assessment of methods to prevent and treat human norovirus infections. Initially, Ettayebi et al. (2016) demonstrated successful *ex vivo* human intestinal enteroid cultures that supported human norovirus replication. They found that replication was restricted to the small intestine based on viral replication in duodenal and ileal HIEs, but not colonoid HIEs. They further optimized new medium conditions to enhance human norovirus cultivation in HIEs (Ettayebi et al., 2021). Another study evaluated virus inactivation strategies using the HIEs model. Alcohols were found to slightly reduce but not completely inactivate human norovirus, regardless of concentration or exposure time. In contrast, complete inactivation of GII.4 viruses occurred at concentrations as low as 50 ppm of chlorine (Costantini et al., 2018). Moreover, another study showed that aged green tea extract could be an effective natural compound against human norovirus GII.4 through HIE cultures (Randazzo et al., 2020). Additionally, one study evaluated the correlation between viral load measured by real-time reverse transcription PCR and virus infectivity through HIE cultures. Norovirus inocula with a cycle threshold value <30 were found to robustly yield productive virus replication in HIEs, indicating the presence of infectious virus (Chan et al., 2019).

Recently, there have been more studies investigating the host-pathogen interaction of norovirus using human intestinal enteroids (HIEs) cultures. In one study, the role of FUT2, a gene involved in glycosylation, in human norovirus replication was evaluated. The results showed that FUT2 expression affected both the binding of human norovirus to the HIE cell surface and the susceptibility to viral infection, indicating that binding to a molecule glycosylated by FUT2 was critical for human norovirus infection (Haga et al., 2020). Another study identified a predominant type III interferon (IFN)-mediated innate response to human norovirus infection in HIE cultures. The study further revealed strain-specific sensitivities to IFN, showing that in signal transducer and activator of transcription 1 (STAT-1)-knockout HIEs compared to parental HIEs, there was enhanced replication and virus spread for GII.3, instead of the globally dominant GII.4 human norovirus. This indicated that IFN restricted GII.3 but not GII.4 replication, which might explain why GII.4 infections were more widespread and pandemic (Lin et al., 2020). Furthermore, another study showed that GII.3 replication in HIEs was dependent on bile acids. Glycochenodeoxycholic acid (GCDCA) induced multiple cellular responses that promoted GII.3 replication in HIEs, including endosomal uptake enhancement, endosomal acidification, and subsequent activation of endosomal/lysosomal enzyme acid sphingomyelinase (ASM). Inhibitors of endosomal acidification or ASM reduced GII.3 infection, but exogenous addition of ceramide alone permitted infection (Murakami et al., 2020). Therefore, HIE cultures provide an *ex vivo* model for studying human norovirus and have been valuable in uncovering important insights into host-pathogen interactions, and strain-specific sensitivities to host immune responses.

## 4. Effect of norovirus on microbiota profiles

There have been several clinical studies that have reported changes in microbiota profiles following norovirus infections in infants and adults (Table 1). Infants infected with human norovirus were found to have higher abundance of *Fusobacteria* and *Cyanobacteria* at the phylum level, and higher abundance of *Bacillus* spp., *Enterococcus spp.*, and *Streptococcus spp.* at the genus level compared to healthy infants. The Chao1 index, which indicates microbial diversity, was significantly higher in the human norovirus group, and there were significant differences in potentially pathogenic bacteria (Xiong et al., 2021). Infants with positive norovirus test results for diarrhea had significantly higher counts of *Enterobacter cloacae* compared to those who tested negative for norovirus (Magwira et al., 2021). Another study analyzed stool samples from infants and found that the microbiome was dominated by *Actinobacteria* before infection, with no change during norovirus infection episodes. However, there was a shift with a higher proportion of *Bacilli* and *Clostridia* observed several weeks after the first episode of acute gastroenteritis ended (Cannon et al., 2022). Fecal microbiome analysis from pediatric patients with severe norovirus or rotavirus infection revealed a decreased Shannon diversity index of the intestinal microbiota, and a greater abundance of *Campylobacteraceae*, *Neisseriaceae*, *Methylobacteriaceae*, *Sphingomonadaceae*, and Enterobacteriaceae compared to healthy children (Chen et al., 2017). Furthermore, the abundance of *Bifidobacterium* spp. and *Lactobacillus* spp. was significantly reduced in children with norovirus or rotavirus infection compared to healthy children (Solano-Aguilar et al., 2013).

**Table 1:** The relationship between norovirus and gut microbiome.

Intestinal microbiota changes		Interaction mechanism	
Infants	Adults	Anti-viral effects	Pro-viral effects
Higher *Fusobacteria* and *Cyanobacteria* Higher *Bacillus spp.*, *Enterococcus* spp. and *Streptococcus spp.* (Xiong et al., 2021)	Higher abundance of *Escherichia-Shigella* (Mizutani et al., 2021)	*Lactobacillus* and γ-PGA from *Bacillus spp.* upregulated IFN-β against murine norovirus (Lee and Ko, 2016; Lee et al., 2018)	HBGA expressed by microbiota could bind to viral capsid and enhanced cell attachment and environmental stability (Miura et al., 2013; Jones et al., 2014; Li et al., 2015; Budicini and Pfeiffer, 2022)
Higher *Enterobacter cloacae* (Magwira et al., 2021)	Decreased numbers of *Bacteroidetes*; increased *Proteobacteria* (Nelson et al., 2012)	Murine astrovirus induced IFN-λ and bacteriophage induced IFN-γ against murine norovirus (Yokoyama et al., 2012; Ingle et al., 2019; Zhang L. et al., 2021)	Bile acid could enhance HBGA binding and essential for viral growth and escape from endosomes (Chang et al., 2004; Shivanna et al., 2014; Kilic et al., 2019)
Higher proportion of *Bacilli* and *Clostridia* (Cannon et al., 2022)	Increased numbers of *Firmicutes* and decreased *Bacteroidetes* and *Proteobacteria* in symptomatic groups (Patin et al., 2020)	Priming of IFN-λ response by bacteria-biotransformed bile acids to block murine norovirus replication (Sayin et al., 2013; Grau et al., 2020)	Metabolite triggered type 2 cytokines which could induce tuft cell proliferation and promote murine norovirus *in vivo* (Lei et al., 2018; Nadjsombati et al., 2018; Wilen et al., 2018)
Higher abundance of *Campylobacteraceae*, *Neisseriaceae*, *Methylobacteriaceae*, *Sphingomonadaceae*, and *Enterobacteriaceae* (Chen et al., 2017)	*Pseudomonas* genus dominant in symptomatic groups *Bacteroides, Erwinia, Agrobacterium* genera and *Ruminococcaceae* family in asymptomatic groups (Mori et al., 2018)	OMVs led to higher pro-inflammatory cytokines (Bhar et al., 2022; Mosby et al., 2022, 2023)	Counteracted IFN-λ pathway and induced higher sIg (Turula et al., 2018)

γ-PGA, poly-γ-glutamic acid; IFN-β, interferon-beta; IFN-λ, interferon-lambda; IFN-γ, interferon-gamma; OMVs, outer membrane vesicles; HBGA, histo-blood group antigen; sIg, secretory immunoglobulins.

Statistical differences were observed in the microbiota profiles of healthy adults and adult patients with diarrhea, with *Firmicutes*, *Actinobacteria*, *Bacteroides*, *Cyanobacteria*, and *Proteobacteria* showing significant differences. In healthy adults, *Faecalibacterium* was the dominant genus, while in patients with diarrhea, *Escherichia-Shigella* was the most abundant (Mizutani et al., 2021). Some norovirus-infected patients showed significant alterations in their microbiota compared to uninfected individuals, characterized by reduced relative numbers of *Bacteroidetes* and an increase in *Proteobacteria* due to a single operational taxonomic unit (OTU) of *Escherichia coli.* Factors such as gender, age, lactoferrin levels, and sampling time did not explain these changes in the microbiota (Nelson et al., 2012). Symptomatic individuals infected with norovirus exhibited a marked shift in their microbiome compared to asymptomatic individuals, with an increase in *Firmicutes* immediately after infection and a decrease in *Bacteroidetes* and *Proteobacteria* over the same time period. Genes enriched in the microbiomes of symptomatic subjects, such as the adenylyltransferase glgC, were associated with glycan metabolism and cell–cell signaling (Patin et al., 2020). Another study comparing symptomatic individuals with asymptomatic carriers found that the *Pseudomonas* genus was dominant in symptomatic groups, while *Bacteroides*, *Erwinia*, *Agrobacterium* genera, and *Ruminococcaceae* family were dominant in asymptomatic carriers (Mori et al., 2018). Studies on murine norovirus showed that malnutrition and murine norovirus infection could heavily influence the gut microbial composition, especially a decreased *Bacteroidetes/Firmicutes* ratio was observed (Hickman et al., 2014). However, another study found no major differences in intestinal bacterial communities between murine norovirus-infected mice and uninfected controls, both in tissue-associated samples and feces (Nelson et al., 2013).

## 5. Effects of microbiota on norovirus

### 5.1. Antiviral effects

Several studies have reported certain commensal bacteria play an important role in host susceptibility to norovirus infection. A higher abundance of *Ruminococcaceae* and *Faecalibacterium* bacteria was correlated with lower anti-viral immunoglobulin A (IgA) titers, suggesting that individuals with these taxa may have lower susceptibility to human norovirus infection (Rodriguez-Diaz et al., 2017). In a mouse model, human norovirus genotype GII.4 efficiently replicated in antibiotic-treated mice with depleted microbiota. Genera such as *Adlercreutzia*, *Ruminococcus*, and *Dorea* were depleted after antibiotic treatment, resulting in low microbial diversity, which was linked to increased permissiveness of norovirus replication. The expression of interleukin-4 (IL-4) and IL-13 was upregulated in all antibiotic-treated groups, but the expression levels of toll-like receptor 2 (TLR2) and tumor necrosis factor alpha (TNF-α) were diminished, resulting in efficient replication of norovirus (Santiso-Bellón et al., 2022).

Most of the following studies explored antiviral effects including interferon (IFN) modulation and increased production of smaller outer membrane vesicles (OMVs) from gut microbiome to block murine norovirus infection (Table 1). Firstly, both commensal bacteria-derived ligands and metabolites can signal and regulate IFN signaling pathways (Wirusanti et al., 2022). In an immunocompetent mouse model, STAT-1-dependent IFN responses restricted murine norovirus infection by inhibiting viral replication in the intestine, preventing virus-induced apoptosis of intestinal cells and splenocytes, and limiting viral dissemination to peripheral tissues (Mumphrey et al., 2007). Poly-γ-glutamic acid (γ-PGA), an extracellular biopolymer produced by *Bacillus* spp., was identified as a non-canonical toll-like receptor 4 (TLR4) agonist with anti-viral effects against norovirus infection. In *ex-vivo* culture of mouse ileum, oral administration of γ-PGA increased serum IFN-β levels, reduced murine norovirus loads in the ileal Peyer’s patches (PPs), and mesenteric lymph nodes in mice (Lee et al., 2018). Another *in vivo* and *in vitro* model showed that the *Lactobacillaceae* families were remarkably increased by murine norovirus inoculation and retinoic acid administration. Upregulated IFN-β by the abundance of *Lactobacillus* showed anti-viral effects against murine norovirus (Lee and Ko, 2016). A clinical study conducted with a similar concept showed the effect of retinoic acid supplementation on human norovirus infection among young children. Retinoic acid administration reduced the prevalence of human norovirus GII infections, increased the length of viral shedding, and decreased the prevalence of diarrhea (Long et al., 2007).

Next, research has shown that the virome, including bacteriophages, can have anti-viral effects. In immunodeficient hosts, the virome can protect against enteric pathogens. For instance, murine astrovirus has been observed in immunodeficient mice such as Rag1(−/−) mice, which are deficient in B and T cells (Yokoyama et al., 2012). The presence of murine astrovirus (STL5) in the gut has been found to protect primary immunodeficient mice from murine norovirus infection through IFN-λ signaling in gut epithelial cells. This protection can also be horizontally transferred between immunocompromised mice through co-housing and fecal transplantation (Ingle et al., 2019). IFN-λ has been identified as a key cytokine for curing virus infections. While cytokines such as IFN-α and IFN-β can prevent the systemic spread of murine norovirus, only IFN-λ can effectively control persistent enteric infections. The induction of IFN-λ is regulated by the murine norovirus capsid protein and is correlated with diminished enteric persistence (Nice et al., 2015). In addition, another study investigated the role of bacteriophages in murine norovirus replication. The results demonstrated that bacteriophages increased cellular response to IFN-γ and IFN-inducible GTPases, which exerted an antiviral effect *in vitro* (Zhang L. et al., 2021).

Finally, the interactions between norovirus and bacteria also had an impact on the bacteria themselves. These interactions induced stress responses in the bacteria and increased the production of bacterial extracellular vesicles. Specifically, when norovirus interacted with commensal bacteria such as *Enterobacter cloacae*, *Bacteroides thetaiotaomicron*, and *Lactobacillus acidophilus*, it led to an increased production of smaller OMVs. The stool samples collected from mice infected with the virus showed a significant increase in vesicle production compared to mock-infected controls (Mosby et al., 2022). Norovirus infection also resulted in changes in the DNA, protein, and lipid content of the bacteria. The increased accumulation of phospholipids was associated with increased blebbing, but there was no change in the lipid content of the bacterial outer membrane or the metabolite content of the bacterial cell (Mosby et al., 2023). Additionally, another study showed that murine norovirus could attach to the OMVs, facilitating co-inoculation of target cells with both the virus and vesicles. When murine noroviruses and OMVs were co-inoculated into macrophages, viral infection was reduced. Co-inoculation of murine norovirus with OMVs resulted in higher production and release of pro-inflammatory cytokines (IL-1β, TNF-α, and IFN-β) in response to viral infection compared to murine norovirus alone (Bhar et al., 2022).

### 5.2. Pro-viral effects

Gut microbiota may facilitate norovirus replication through various mechanisms, such as binding of the virus to HBGAs expressed by both the host and certain bacteria, bile acid-associated immune response, and tuft cells induced by type 2 cytokine (Table 1). Some human microbiota, such as *Enterobacter cloacae*, *Escherichia coli*, and *Helicobacter pylori*, have been shown to express HBGAs (Rasko et al., 2000; Yi et al., 2005; Jones et al., 2014), and human norovirus can bind to these bacteria. Both probiotic and non-probiotic bacteria have been found to possess the ability to bind to norovirus genotypes GI.1 and GII.4 through the protruding (P)-domain of the norovirus VP1 capsid protein (Rubio-del-Campo et al., 2014). Studies have shown that the extracellular polymeric substances (EPS) of *Enterobacter cloacae* containing HBGA-like substances play a key role in binding with human norovirus (Miura et al., 2013). Moreover, human norovirus has been found to enhance its infectivity to host cells in the presence of HBGA-positive bacteria by binding viral particles to these bacteria, allowing uptake of the virus into host cells (Jones et al., 2014). HBGA-positive bacteria are likely to affect the transmission and infection process of human norovirus. For instance, HBGA-positive *Escherichia coli* has been shown to maintain higher mucin-binding ability of norovirus-like particles even after heat treatment (Li et al., 2015). Additionally, both gram-positive and gram-negative bacteria have been found to bind to murine norovirus, and virion stability was enhanced in the presence of several gram-positive bacterial strains due to small heat-stable molecules (Budicini and Pfeiffer, 2022). Interestingly, human norovirus-bacteria binding has been observed around the outer cell surfaces and pili structures of gut microbiota, even if they do not produce HBGA (Almand et al., 2017). Other carbohydrate moieties, such as terminal sialic acids, glycoprotein, glycolipid, or glycosaminoglycans, have also been reported as receptors for both murine (Taube et al., 2009, 2012) and human norovirus (Tamura et al., 2004; Rydell et al., 2009). In terms of environmental stability, enteroviral particles, including norovirus, were packaged within vesicles enriched with phosphatidylserine (PS) lipids. These vesicles were released from cells without causing lysis and facilitated greater infection efficiency through bacterially-mediated viral clustering (Chen et al., 2015). These vesicles, which enveloped virus clusters, remained intact during fecal-oral transmission, allowing for the transport of multiple viral particles to the next hosts and enhancing viral tolerance to extreme temperatures, pH levels, and salt concentrations (Santiana et al., 2018). The influence of these vesicles on viral interaction with bacteria has yet to be explored, but it presents an interesting topic for future research.

Furthermore, it has been found that bile acid is required for certain genotypes of human norovirus (Ettayebi et al., 2016). Bile acid enhances the binding of HBGA for the HBGA binder genotype GII.10, and the non-HBGA binder genotype GII.1 can be converted to a HBGA binder after bile acid binding. Human norovirus genotypes GII.4 and GII.17, which do not bind bile acid, are responsible for large epidemics, whereas genotypes GII.1 and GII.10, which do bind bile acid, are not as widespread, indicating that these viruses have modified their bile acid requirements on the capsid (Kilic et al., 2019). In studies on porcine enteric calicivirus, bile acid was found to be critical for viral escape from the endosomes, enabling entry into the cell cytoplasm for viral replication (Shivanna et al., 2014). Bile acid was also found to be essential for viral growth and was associated with the down-regulation of IFN-mediated STAT-1 phosphorylation (Chang et al., 2004). Bile acids are synthesized from cholesterol in the liver and further metabolized by gut microbiota into secondary bile acids. Intestinal anaerobic bacteria also carry out bile salt hydrolysis, hydroxy group dehydrogenation reactions, and other biotransformation processes (Ridlon et al., 2006). The bile acid receptor farnesoid X receptor (FXR) plays a role in regulating both pro-viral and antiviral capacities. Bile acid synthesis is under negative feedback control through activation of the nuclear receptor FXR in the ileum and liver. High levels of FXR suppress bile acid-mediated enhancement of IFN-λ expression. One *in vitro* study showed that gut microbiota may suppress cholesterol 7α-hydroxylase (CYP7A1) and bile acid synthesis by reducing the levels of Tauro-beta-muricholic Acid (a natural FXR antagonist) and promoting FXR-dependent fibroblast growth factor 15 (FGF15) expression in the ileum (Sayin et al., 2013). Another *in vitro* study revealed that gut microbiota has opposing regional effects on murine norovirus infection, inhibiting viral infection of the proximal gut while promoting viral infection of the distal gut. The inhibition of proximal gut infection was due to the priming of IFN-λ response by bacteria-biotransformed bile acids, which in turn blocked viral replication. However, the promotion of distal gut infection was due to the more abundant FXR expression in the distal gut (Grau et al., 2020). Therefore, bile acid is necessary for certain genotypes of human norovirus, and the immune response associated with bile acid may be controlled by gut microbiota.

Furthermore, CD300lf, a proteinaceous receptor, was found to be essential for the binding and replication of murine norovirus. CD300lf-deficient mice [Cd300lf (−/−)] showed resistance to murine norovirus infection, indicating that CD300lf was the primary determinant of murine norovirus species tropism (Orchard et al., 2016). The dimeric protruding (P) domain of murine norovirus VP1 was found to form a complex with its cellular receptor CD300lf. The P2 subdomain, which has a cleft between the AB and DE loops, overlaps with the epitopes of neutralizing antibodies (Nelson et al., 2018). The structure of CD300lf-P domain complexed with glycochenodeoxycholic acid (GCDCA) and lithocholic acid (LCA) revealed that GCDCA induced conformational changes in the P domain, resulting in the elimination of P domain recognition by neutralizing antibodies as a viral immune escape mechanism (Creutznacher et al., 2021). Tuft cells, a rare type of intestinal epithelial cell, were found to express CD300lf and serve as the target cell for murine norovirus in the mouse intestine. Type 2 cytokines, including IL-4, which induce tuft cell proliferation, were found to promote murine norovirus infection *in vivo* (Wilen et al., 2018). Moreover, both succinate receptor 1 (Sucnr1) expressed on tuft cells and the microbial metabolite succinate, as an activating ligand, may trigger type 2 immunity (Lei et al., 2018; Nadjsombati et al., 2018).

Moreover, several studies have demonstrated that the bacterial microbiome can play a role in fostering enteric viral persistence by mediating the IFN-λ immune pathway. Antibiotics were found to prevent persistent murine norovirus infection by targeting IFN-λ receptor 1 (IFNLR1), the receptor for the antiviral cytokine IFN-λ, as well as the transcription factors STAT-1 and IFN regulatory factor 3 (IRF3). This prevention was reversed by replenishment of the bacterial microbiota (Baldridge et al., 2015). Heme-oxidized IRP2 ubiquitin ligase 1 (HOIL1) was shown to be critical for type I and III IFN induction during murine norovirus infection, as Hoil1(−/−) mice exhibited defective control of murine norovirus infection. Similarly, defective regulation of infection by the microbiome was observed in mice deficient in IFNLR1, STAT-1, and IRF3 (MacDuff et al., 2018). The normal microbiome was found to induce higher levels of secretory immunoglobulins (sIg) and promote murine norovirus infection. In mice lacking the polymeric immunoglobulin receptor (pIgR) relative to control mice, increased levels of IFN-γ and inducible nitric oxide synthase (iNOS) were observed, accompanied by reduced murine norovirus titers (Turula et al., 2018).

## 6. Effects of probiotics on norovirus

Probiotics have been used to prevent and reduce symptoms of common viruses, including digestive infections such as rotavirus, coronavirus, and norovirus. Various strains of *Lactobacillus* and *Bifidobacterium* have been frequently studied for their potential anti-viral effects (Lopez-Santamarina et al., 2021). Probiotics can modulate the immune response by promoting maturation of immune cells such as intestinal macrophages and dendritic cells. Additionally, probiotics can exert anti-viral activities through mechanisms such as competitive exclusion, bacteriocin production, and stimulation of antimicrobial defenses (Steyer et al., 2022). Probiotics can also protect the gut by increasing mucosal secretion, improving intestinal motility, and enhancing production of short-chain fatty acids (Vitetta et al., 2018). *In vitro* studies have shown that bacterial metabolic products from commercial probiotics containing strains such as *Lactobacillus acidophilus*, *Lactobacillus rhamnosus*, *Bifidobacterium bifidum*, *Lactobacillus salivarius*, and *Streptococcus thermophilus* have inhibitory effects on murine norovirus propagation (Adrienne et al., 2014). Other studies have demonstrated that strains such as *Weissella cibaria*, *Pediococcus pentosaceus*, *Lactobacillus curvatus*, and *Lactobacillus sakei* can significantly reduce murine norovirus (Seo et al., 2020), while *Bifidobacterium adolescentis* can inhibit the multiplication of murine norovirus (Li et al., 2016). Furthermore, a centenarian gut-derived strain, *Limosilactobacillus fermentum*, has been shown to exhibit the strongest antagonism against murine norovirus (Li et al., 2022).

Firstly, *Lactobacillus salivarius HHuMin-U (HHuMin-U)* effectively suppressed the replication of murine norovirus and decreased viral RNA levels in macrophages *in vitro*. *HHuMin-U* activated specific signaling pathways, including nuclear factor κB and TANK-binding kinase 1 (TBK1) - interferon regulatory factor 3 (IRF3), which led to increased production of IFN-α, IFN-β, and TNF-α in mouse macrophages (Kim D. H. et al., 2022). Next, a single-chain variable fragment (scFv) called 3D8, which is secreted by genetically engineered *Lactobacillus paracasei*, prevented apoptosis induced by murine norovirus infection and decreased the expression of viral capsid protein messenger RNA (Hoang et al., 2015). *In vivo* studies, both *Lactobacillus salivarius* and scFv 3D8 from *Lactobacillus paracasei* revealed antiviral effects (Table 2). In another live animal study, *Limosilactobacillus reuteri* strain *Byun-re-01* was found to produce anti-inflammatory cytokines, mainly IFN-β and IFN-γ, at the mRNA level. It also suppressed the expression of viral capsid mRNA of murine norovirus (Kim D. et al., 2022). Furthermore, oral administration of *Lactobacillus johnsonii* strain *Byun-jo-01* showed the highest antiviral efficacy against murine norovirus compared to feeding with other probiotic strains (Kim et al., 2021). In a study using a gnotobiotic pig model, colonization with *Lactobacillus rhamnosus* strain *GG (LGG)* and *Escherichia coli* strain *Nissle 1917 (EcN)* inhibited human norovirus shedding and significantly reduced the incidence and duration of diarrhea. The protective efficacy of these probiotic regimens was attributed to the stimulation of IFN-γ + T cell responses, increased production of intestinal IgA and IgG, and maintenance of healthy intestinal morphology (Lei et al., 2016).

**Table 2:** Effects of probiotics against norovirus.

Type of study	Probiotics	Dosage and time	Viruses	Findings	Reference
*In vivo* study of mice	*L. salivarius HHuMin-U*	3 × 10^10^ CFU/kg of body weight once daily for 5 days	Murine norovirus	Enhanced the expression level of antiviral IFN-α and IFN-β and TNF-α production	Kim D. H. et al. (2022)
*In vivo* study of 12 mice	*L. paracaseiatcc334*	10^8^ CFU daily for 6 days	Murine norovirus	Reduced mRNA that encoded viral polymerase	Hoang et al. (2015)
*In vivo* study of 12 mice	*Limosilactobacillus reuterii Byun-re-01; Limosilactobacillus reuteri KACC 11452; L. paracasei ATCC 334*	10^8^ CFU daily for 2 weeks	Murine norovirus	Produced IFN-β and IFN-γ in mRNA levels; Suppressed the expression of viral capsid mRNA	Kim D. et al. (2022)
*In vivo* study of 12 mice	*L. johnsonii Byun-jo-01; L. paracasei ATCC 334; Limosilactobacillus reuteri KACC 11452*	10^8^ CFU daily for 2 weeks	Murine norovirus	*L. johnsonii Byun-jo-01* showed the highest antiviral efficacy	Kim et al. (2021)
*In vivo* study of 28 gnotobiotic pigs	*L. rhamnosus GG + Escherichia coli Nissle 1917*	10^4^ CFU of each mixed in five ml medium every 2 days	Human norovirus GII.3	Inhibited viral shedding and reduced the mean duration of diarrhea; Stimulated IFN-γ response	Lei et al. (2016)
Case-controlled study of 77 elderly	*L. casei strain Shirota (LcS-fermented milk)*	4 × 10^10^ cells/80 mL bottle for 2 weeks	Human norovirus	Shorter mean duration of fever, *Bifidobacterium* and *Lactobacillus* were significantly dominant from feces	Nagata et al. (2011)
Double blind placebo-controlled trials of 150 children	*L. acidophilus*	2 × 10^8^ CFU twice daily for 5 days	Human norovirus	Do not shorter the time to the start of the first 24-h period without diarrhea	Hong Chau et al. (2018)
Double blind placebo-controlled trials of 816 children	*L. rhamnosus R0011 + L. helveticus R0052*	4 × 10^9^ CFU of a *L. rhamnosus* R0011 and *L. helveticus R0052* (95:5 ratio) twice daily for 5 days	Adenovirus, norovirus, and rotavirus	Do not reduce the severity of acute gastroenteritis or expedite the clearance of viruses in stool	Freedman et al. (2020)

CFU, colony forming unit; IFN-α, interferon-alpha; IFN-β, interferon-beta; TNF-α, tumor necrosis factor-alpha; IFN-γ, interferon-gamma; mRNA, messenger RNA.

In a clinical trial, a case-controlled study examined the effects of probiotic-fermented milk containing *Lactobacillus casei* strain *Shirota* in elderly individuals with norovirus gastroenteritis. The results showed that the mean duration of fever was significantly reduced in the group that received the probiotic. Analysis of fecal samples also revealed a significant increase in *Bifidobacterium* and *Lactobacillus,* while *Enterobacteriaceae* decreased in the probiotic group (Nagata et al., 2011). However, a double-blind, placebo-controlled trial investigating the effects of probiotic treatment containing *Lactobacillus acidophilus* on children with acute norovirus or rotavirus infection found no significant difference in the time to the start of the first 24-h period without diarrhea, indicating no benefits of probiotics in treating acute watery diarrhea in children (Hong Chau et al., 2018). Another double-blind, placebo-controlled trial involving 816 children with acute gastroenteritis also showed no virus-specific beneficial effects of the probiotic in reducing viral nucleic acid clearance from stool specimens (Freedman et al., 2020). Furthermore, there were no differences in diarrhea duration or the total number of diarrheal stools between the treatment and placebo groups across various common acute gastroenteritis pathogens, including adenovirus, norovirus, rotavirus, and bacteria (Freedman et al., 2021).

## 7. Norovirus vaccine development

The development of a norovirus vaccine has been challenging due to the wide genetic and antigenic diversity of circulating norovirus strains. Additionally, there have been limitations in culture systems, animal models, and immune markers for vaccine evaluation (Tan, 2021). In recent years, several approaches have been explored for developing a norovirus vaccine, as summarized by Lucero et al. (2021). These vaccine platforms include virus-like particles (VLPs), P-particles, and adenovirus vector-based vaccines. Norovirus VLPs have been expressed in *Escherichia coli* (Tan et al., 2004; Huo et al., 2018), yeast (Tome-Amat et al., 2014; Parker et al., 2016) and plants (Mathew et al., 2014). Immune responses induced by these vaccines tend to be genotype-specific, requiring multiple genotypes in the vaccine formulation. Most of the vaccine candidates are in the pre-clinical stage, with some already reported in clinical trials. Clinical trial registrations were searched from https://clinicaltrials.gov/. Five vaccine candidates from Takeda, Vaxart, National Vaccine and Serum Institute China (NVSI), Anhui Zhifei Longcom Biologic Pharmacy, and Icon Genetics GmbH have been studied in either phase 1 or phase 2 clinical trials, with most of the studies complete and some reporting safety and efficacy results. The vaccine from Takeda, which contains two types of virus-like particles from GI.1 and GII.4 strains, is the most extensively studied. Several phase 1 clinical trials (El-Kamary et al., 2010; Treanor et al., 2014; Bernstein et al., 2015; Sundararajan et al., 2015) have demonstrated that the vaccine is well tolerated and induces robust immune responses in healthy adults. Subsequent phase 2 clinical trials (Atmar et al., 2016, 2019; Leroux-Roels et al., 2018; Masuda et al., 2018; Treanor et al., 2020) further confirmed the efficacy of the vaccine in participants ranging from 6 months old to older than 60 years old, with one study (Sherwood et al., 2020) even showing significant efficacy against all types of norovirus acute gastroenteritis, indicating cross-genotype protection. Table 3 provides information on the antigen genotypes and adjuvants used in the five vaccine candidates, as well as clinical trial information such as participant age, vaccine formulations, and published results.

**Table 3:** Five norovirus vaccine under clinical trials.

Company	Vaccine	Antigen format antigen genotype	Adjuvant	Reference/Clinical trial^†^
Takeda	HIL-214 (TAK-214) (IM or IN)	VLPs in baculovirus system GI.1/GII.4 GI.1: prototypic Norwalk virus GII.4: 3 viruses, 2006a (Yerseke), 2006b (Den Haag), and 2002 (Houston)	Chitosan/MPL/Aluminum	(El-Kamary et al., 2010) NCT00806962 (p1, age 18–49, monovalent GI.1, intranasal, Chitosan/MPL; tolerated) (Treanor et al., 2014) NCT01168401 (p1, age 18–49, bivalent, MPL/Aluminum; rapid response after single dose, tolerated) (Bernstein et al., 2015; Sundararajan et al., 2015) NCT01609257 (p1-2, age 18–50, bivalent, MPL/Aluminum; reduced vomiting/diarrhea; single dose effective for B cell memory) (Leroux-Roels et al., 2018) NCT02038907 (p2, age 18–64, bivalent, Aluminum+/-MPL; 15μgGI.1/ 50 μg GII.4c best immune, MPL no benefit) (Atmar et al., 2016, 2019) NCT02142504 (p2, age 18–49, bivalent, MPL/Aluminum; 15μgGI.1/ 50 μg GII.4c best immune, persist at least 1 year) (Masuda et al.) NCT02153112 (p2, age 0.5–4, bivalent, Aluminum; robust immune, highest response after 2 doses of the 50/150 μg) (Treanor et al., 2020) NCT02661490 (p2, age > = 60, bivalent, Aluminum+/-MPL; safe, immune response unaffected by age or MPL) (Sherwood et al., 2020) NCT02669121 (p2b, age 18–49, bivalent, Aluminum; cross-genotype protection, against any norovirus AGE) (Vesikari et al., 2022) (age 1–8, bivalent, Aluminum; tolerated, increased immune response after second dose)
Vaxart	VXA-G1.1-NN (Oral) VXA-GII.4-NS (Oral)	Nonreplicating recombinant adenovirus vector encodes VP1 GI.1/GII.4 GI.1: Norwalk strain GII.4: Sydney variant	Adenovirus dsRNA	(Kim et al., 2018) NCT02868073 (p1, age 18–49, adenovirus-based monovalent GI.1 VP1, dsRNA; tolerated, oral vaccine) NCT03125473 (p1b, 66 participants, age 18–49, adenovirus-based monovalent GI.1 VP1, dsRNA) NCT03897309 (p1b, 86 participants, age 18–49, adenovirus-based monovalent or bivalent of GI.1/GII.4, dsRNA) NCT04854746 (p1b, 66 participants, age 55–80, adenovirus-based monovalent GI.1 VP1, dsRNA) NCT04875676 (p1b, 30 participants, age 18–55, adenovirus-based monovalent GI.1 VP1, dsRNA) NCT05213728 (p1, 8 participants, age 18–55, adenovirus-based monovalent GI.1 VP1, dsRNA) NCT05626803 (p2, 135 participants, age 18–80, adenovirus-based bivalent GI.1/GII.4, dsRNA)
NVSI	Hansenula Polymorpha (IM)	VLPs in Hansenula polymorpha system GI.1/GII.4	Aluminum	NCT04188691 (p1, 450 participants, age 0.5–59, bivalent, Aluminum) NCT04941261 (p2, 1716 participants, age 0.5–59, bivalent, Aluminum)
Anhui Zhifei Longcom	Longkoma (IM)	VLPs in Pichia pastoris system GI.1/GII.3/GII.4/GII.17	Aluminum	NCT04563533 (p1/2a, 580 participants, age > 6 weeks, quadrivalent, Aluminum)
Icon Genetics GmbH	rNV-2 V (IM)	VLPs in Green plant system GI.4/GII.4 GI.4: Chiba 407 (1987) GII.4: Aomori 2 (2006)	No adjuvant	(Leroux-Roels et al., 2022) NCT05508178 (p1, age 18–40, bivalent, no adjuvant, tolerated, peak immune of 50 μg GI.4 + 50 μg GII.4 after Day 8–28)

IM, intramuscular, IN, intransal; MPL, monophosphoryl lipid A; VLP, virus like particles; dsRNA, double strand RNA; p1, phase 1; p2, phase 2.

^†^Published: Reference/Clinical trial number (phases of trials, enrolled age, vaccine formulation; published results). Haven’t published: Clinical trial number (phase of trials, enrolled participants number, age, vaccine formulation).

## 8. Discussion and future direction

The microbiota profile changes and the interaction between microbiota and norovirus infection were recently observed and highlighted in this review. In addition, this review summarizes the updated *in vivo* and clinical studies of probiotics on norovirus infection and investigates the clinical trials and published results of five vaccine candidates. This review deepens our understanding of the relationship between microbiota and norovirus infection, providing a powerful tool for the treatment of norovirus infections and the development of antiviral strategies, including probiotics or norovirus vaccines in the future.

There is a lot of ongoing research on host glycobiology which could potentially play a role in the outcome of norovirus infection. However, the inherent complexity of host glycobiology and microbiota complicates drawing definitive conclusions. Future studies investigating the interaction between host and pathogen using human intestinal enteroids system that mimic human intestinal epithelium may overcome this challenge. For instance, enteroids system generated from individuals with different secretor status may provide information about the relationship between host glycobiology and norovirus infection. The recent discovery of noroviruses potentially being packed within extracellular vesicles and the effect of gut microbiota further complicates the study of viral pathogenesis and inactivation. The role of outer membrane vesicles and bile acid-associated immune response controlled by bacteria also remains an area of interest for further study. Moreover, the virome, the viral fraction of the microbiome in the intestinal microbiome, requires further investigation of its interaction with norovirus infection.

Regarding antiviral strategies, probiotics have been studied for their antiviral effects in recent years; however, the specific immune mechanisms remain unclear. In addition to *in vivo* studies, clinical trials studying the antiviral effects of probiotics on individuals with norovirus infection are needed. Furthermore, challenges need to be addressed for norovirus vaccine development. Although young children experience the highest incidence of disease and severe disease outcomes are most common among young children and the elderly, most vaccine clinical studies have focused on adults. Separate clinical development for adults and children is required to determine the timing and number of doses, antigen concentration, and the need for adjuvants. Phase III clinical studies are necessary to demonstrate efficacy against natural infection in the field. Moreover, a multivalent vaccine that targets several pathogens in a single dose may be more attractive than introducing additional single-target vaccines; however, the role of these different groups in transmission and the transmission-blocking potential of a vaccine should be better understood. Lastly, testing the efficacy of antiviral strategies, including HBGAs and gut microbiota, in relation to host glycobiology and microbiota will be necessary due to their important role in norovirus infection.

## Author contributions

G-HB and M-CT wrote the draft manuscript. S-CL, Y-HH, and S-YC reviewed and edited the manuscript. All authors have read and agreed to the published version of the manuscript.

## Funding

The study was supported by grants from NSTC 112-2622-E-038-004, Taiwan.

## Conflict of interest

The authors declare that the research was conducted in the absence of any commercial or financial relationships that could be construed as a potential conflict of interest.

## Publisher’s note

All claims expressed in this article are solely those of the authors and do not necessarily represent those of their affiliated organizations, or those of the publisher, the editors and the reviewers. Any product that may be evaluated in this article, or claim that may be made by its manufacturer, is not guaranteed or endorsed by the publisher.
