# Corrigendum: Unraveling the interplay between norovirus infection, gut microbiota, and novel antiviral approaches: a comprehensive review

**DOI:** 10.3389/fmicb.2023.1324539

**Published:** 2023-12-01

**Authors:** Geng-Hao Bai, Meng-Chen Tsai, Sheng-Chieh Lin, Yi-Hsiang Hsu, Shih-Yen Chen

**Affiliations:** ^1^Department of Internal Medicine, National Taiwan University Hospital, College of Medicine, National Taiwan University, Taipei, Taiwan; ^2^Department of General Medicine, Taipei Medical University Hospital, Taipei, Taiwan; ^3^Department of Pediatrics, School of Medicine, College of Medicine, Taipei Medical University, Taipei, Taiwan; ^4^Department of Pediatrics, Division of Allergy, Asthma and Immunology, Shuang Ho Hospital, New Taipei, Taiwan; ^5^Beth Israel Deaconess Medical Center and Harvard Medical School, Boston, MA, United States; ^6^Broad Institute of MIT and Harvard, Cambridge, MA, United States; ^7^Department of Pediatrics, Division of Pediatric Gastroenterology and Hepatology, Shuang Ho Hospital, New Taipei, Taiwan; ^8^TMU Research Center for Digestive Medicine, Taipei Medical University, Taipei, Taiwan

**Keywords:** norovirus, microbiota, probiotics, norovirus vaccine, human intestinal enteroid

In the published article, there was an error in the **Funding** statement. The statement previously read “The study was supported by grants from MOST 108-2314- B-038−097 and MOST 111-2622-E-038-001, Taiwan.” The correct **Funding** statement appears below.

